# Platelet/leukocyte ratio as a predictor of mortality in patients with sepsis

**DOI:** 10.1186/cc12952

**Published:** 2013-11-05

**Authors:** Bárbara Magalhães Menezes, Fábio Ferreira Amorim, Adriell Ramalho Santana, Felipe Bozi Soares, Fernanda Vilas Bôas Araújo, Jacqueline Rodrigues de Carvalho, Mariana Pinheiro Barbosa de Araújo, Louise Cristhine de Carvalho Santos, Pedro Henrique Gomes Rocha, Jaqueline Lima de Souza, Mateus Gonçalves Gomes, Pedro Nery Ferreira Júnior, Alethea Patrícia Pontes Amorim, Rodrigo Santos Biondi, Rubens Antônio Bento Ribeiro

**Affiliations:** 1 Escola Superior de Ciências da Saúde, Brasília, Brazil; 2 Liga Acadêmica de Medicina Intensiva de Brasília, Brazil; 3 Hospital Anchieta, Brasília, Brazil

## Background

The platelet count is an established index in the evaluation of severity in patients with sepsis, and therefore is a component of the SOFA score [1]. Furthermore, the alterations in leukocyte count are also used in the definition of SIRS. The present study aims to evaluate the accuracy of the platelet/leukocyte ratio (P/L) as a predictor of mortality in septic patients.

## Materials and methods

Retrospective cohort study conducted on patients admitted to the ICU of Hospital Anchieta, Brasília, DF, Brazil, during 3 years. The patients with sepsis were divided according to P/L as follows: P/L ≥8 group (HPL) or P/L <8 (LPL). The primary outcome was mortality at 4 and 28 days. Accuracy of P/L to predict ICU mortality was measured with the area under the receiver operating characteristic curve.

## Results

In the present study, 195 patients were enrolled, 41% (*n *= 80) with septic shock. Their mean age was 62.8 ± 21.6 years, SAPS 3 was 62.1 ± 15.0, APACHE II was 20.6 ± 8.6, and length of stay in the ICU was 9 ± 11 days. Mortality at 4 days was 10.8% (*n *= 21) and at 28 days was 12.3% (*n *= 24). The groups P/L <8 and P/L ≥8 did not present differences regarding age (59 ± 20 vs. 65 ± 22, *P *= 0.07) and APACHE II (22 ± 9 vs. 20 ± 9, *P *= 0.19). The LPL group had higher SAPS3 (68 ± 18 vs. 59 ± 13, *P *= 0.00). The LPL was significantly associated with mortality in 4 days (18% vs. 7%, *P *= 0.02) and 28 days (19% vs. 9%, *P *= 0.03). The area under the ROC curve of P/L for mortality at day 4 was 0.628 (95% CI 0.498 to 0.757) and at day 28 was 0.613 (95% CI 0.489 to 0.736).

## Conclusions

P/L <8 at admission was associated with higher mortality in 4 and 28 days in patients with sepsis.
